# Transactions of the Medical and Physical Society of Calcutta

**Published:** 1829-10-01

**Authors:** 


### Transactions of the Medical and Physical Society of Calcutta.

I. An Account of an Epidemic Malignant Ulcer, or Hospital Gangrene. By *J. Adam*, M.D.

II. On Gangrenous Ulcer. By *J. Leslie*, Esq.

From the occasional ravages of this destructive malady in military and indeed in civil service, it has justly attracted of late years a good deal of attention. It appears that malignant ulcer is of very frequent occurrence in India, where, as elsewhere, it often takes on an epidemic character, and proves a most intractable and cruel scourge. Dr. Adam, whose paper stands first upon the list, was led, on these accounts, to investigate the subject, and offers the statements to which we shall now direct our own and the public attention, rather with the view of eliciting information from his brethren, than claiming any credit for himself, or his opinions.

"In November, 1818," says Dr. Adam, "I entered upon the medical charge of the artillery division attached to the Nurbudda field force, consisting of a Company of Europeans, a native troop of horse artillery golundauze and other details, amounting in all to between 7 and 800 men. Having been ordered to a distant station on particular duty, soon after assuming charge, I did not rejoin the artillery till the beginning of the following May. At that period, it may be said the troops, in cantonments at Hussingabad, the then head-quarters, were comparatively healthy. This state continued till the rains, when fever, dysentery, and rather numerous cases of Guinea worm (or *Naharooa*) occurred among the European part of my charge. The natives suffered but slightly from the first of these; and were not attacked at all by the others. It was about the middle of October, that the disease now under consideration first made its appearance, contemporaneously with the change of the monsoon, to which in a great measure I attributed its occurrence. In one of the earliest cases which fell under my observation, ulceration had proceeded to a considerable extent; and it was evident that the peculiar characteristic action had existed for some time previously. This sore was described to me to have been originally a mere pimple, or boil, which scarcely attracted notice: that it suddenly became painful, increased in size, and ultimately assumed the foul aspect which it then presented. Other cases were soon afterwards admitted, and in one or two instances it was found to have attacked patients who had been under treatment in hospital for ulcers in various parts of the body. At last not a sore was met with that did not exhibit the gangrenous character. It affected all the above classes of natives under my care indiscriminately, while only one European showed symptoms of the prevailing malady.

"The epidemic was not confined to the artillery division, but occurred also to a considerable extent in the 1st Battalion 8th Regiment Native Infantry, at that time forming part of the force. It appeared remarkable, however, that of the other native troops in cantonments, consisting of two Battalions of infantry, and one Regiment of cavalry, not a man, as far as I could learn, was attacked by it." 134.

The following is a graphic description of the disease as it then appeared, a description which cannot be abridged without injury.

"The first alteration observable in an ulcer attacked by the epidemic was the suppression of the accustomed discharge, and the color of the granulations changing from a reddish to a pale hue. This was quickly succeeded by loss of vitality in the surface of the sore, with an increased action in the parts immediately around it. The sensibility of these became greatly augmented, and an acute burning sensation was substituted for that simple feeling of pain or tenderness common to all healthy granulating surfaces. Fever supervened, and the patient exhibited in his countenance the most urgent distress.

"This suffering aspect formed a striking feature throughout the disease; and the degree of excitement in the ulcer itself, seemed by no means proportioned to the pain, and general disturbance of the system, of which the expression might be deemed an indication. The progress of the sloughing was rapid, but less striking than in common gangrene. The destruction of parts extended equally in all directions, passing deeply into the cellular membrane and muscles, as well as spreading over the skin, being always preceded by a somewhat rounded and reddened, (as far as this change could be discerned in the cutaneous texture of a native,) and slightly everted border, not unlike the sphacelating sore occasionally met with in Lues Venerea. The color of the mortified parts was a dusky grey; and the discharge which exuded from them, or was removed with the dressings, exhibited a similar appearance. But very little humor of any kind was secreted; and it appeared as if a sudden death had seized the parts, without the intervention of the process of suppuration, or the serous effusion observed in ordinary cases of gangrene. The odor arising from the decomposition of the sloughs in the progress of the disease was offensive in the extreme; but at the commencement no peculiar smell was perceptible. As the sloughing advanced, the pulse became quick, and particularly *hard;* the skin hot, and the tongue furred, of a *deep white color*, but not loaded. The general symptoms at this stage were such as mark a high degree of the remittent fever of the natives. The sphacelating process extending its ravages, large pieces of flesh were destroyed, and removed with the scissars, and the muscles and bone underneath became exposed to view: nor did the blood-vessels always escape; and hæmorrhage in one instance occurred to an extent that was truly alarming. In place of the usual sloughs, clots of coagulated blood were now found lying on the dressings, and the surface below pale, and dotted with a few bloody points; which, while they indicated the source of the hemorrhage, by acting as plugs on the open mouths of the vessels, served also for the time to repress it.

"Such was the general course observed in the cases that came under my treatment. In no instance did death ensue, and recovery to health in most of those where the disease was subdued, might be deemed comparatively rapid, and was equally announced in the improved appearance of the sore, and the expression of the patient's countenance. The sloughing process was no sooner arrested, than granulations of a beautiful florid red shot forth from the bottom and sides of the ulcer—sleep, and an appetite for food returned, and all the natural functions were again performed as in health." 137.

Some cases of the disease are briefly detailed, but after the above description of its characters and progress, they need not be inserted here. Some etiological considerations, however, must not be disposed of in so summary a manner. That portion of the valley of the Nurbudda which was occupied by our military posts at the time, is formed by the Vindhya range of hills on one side, and the Gondwana on the other, extending in length through a space of nearly 300 miles. The approach of the hills on either side contracts its breadth in many parts, but, in general, it may be reckoned at from 15 to 20 miles across from one mountain chain to another. The Nurbudda River holds its course through the valley to the northern side, and the country on its banks, with the exception only of the middle part, is every where rude in the extreme, and covered with forests of deep jungle. The soil consists of a dark coarse earth, the product of decomposed trap and amygdaloid, and from the great superabundance of argillaceous matters in its composition, moisture is both greedily absorbed and long retained.

In the dry months the evaporation which necessarily follows must tend to mitigate the heat, and other causes also combine to render the climate of this part of India comparatively mild throughout the year, and peculiarly "raw" in the cold season. The officers' bungalows in the cantonments were built quite close to the river, and the lines of the sepoys extended for a mile and a half along a small elevation or ridge, which gradually sloped to the open parade ground in front. The two extremities of this line were occupied by the 8th N. I, which corps, as has been stated, was attacked by the epidemic, and the horse artillery; whilst about the centre were stationed the artillery hospitals, of which Dr. Adam was in charge. These three situations were the most exposed of any in the cantonments, and after the change of the monsoon, the prevailing wind was received the more directly, and with more effect, than in any other part of the line. To this circumstance Dr. Adam is inclined to attach much importance, as well as to the influence of the same causes which create the general disposition to fevers of the intermittent kind, common to most districts of India on the accession of the cold season. Certain it is, that the only other prevailing disease among the artillery was intermittent fever, generally of the tertian or quotidian type, which yielded readily to bark, and the means had recourse to. In the cases of ulcer, however, it is remarkable that the sloughing character never supervened to the fever, but the fever to the sloughing; and in no one instance did it exhibit any *regular* intermitting character.

"Although, therefore, I feel disposed to attribute the malignancy of the sores, with their consequent constitutional irritation, and the intermittent fever to one and the same cause, namely the supervention of the cold weather, and the change of the wind, which from the mild S. W. suddenly altered to the chilling, and as the sensations certainly indicated, damp N.E.; I do not believe that there was any necessary connection between the two forms of disease, or that either alternated during the period of their prevalence with the other. I am the more anxious that this should be understood, as the beneficial effects of the practice which I ultimately adopted might be ascribed to its removing the fever by some *specific* action, and thus indirectly curing the ulcers.

"On the Europeans, accustomed to a generous diet and warm clothing, the injurious influence of the causes now assigned was scarcely experienced: but in the case of the natives, indifferently clothed and fed, and at the time very wretchedly accommodated both in their lines and at the hospital, their operation must have been exerted with full force, and their effects severely felt accordingly. How far the wind may have brought with it any peculiar noxious impregnation from the jungles over which it blew, such as is generally known by the term miasmata, I cannot pretend to say. I do not deem it necessary even to adduce the probability of such an additional source of the malady, as in my opinion the simpler causes enumerated were quite sufficient for its production; and have been found to give rise to it in other situations." 145.

We now arrive at an important portion of the paper, that in which the treatment is discussed. Stimulating local applications, with the internal use of bark, opium, nitric acid, wine, &c. were successively employed and successively failed. It is a curious fact that the arsenical solution given internally proved of no avail, but that used as a topical means, diluted with one half water, it produced the most happy effects in a case in which it was employed. Whether its success would have been equal upon further trial, could not be put to the proof, as unfortunately the stock of the medicine failed, and no fresh supply could be procured. The charcoal powder and bark appeared to render the ulcers cleaner, but the success of these and of other means would seem to have been trifling at the best. Change of air was now resorted to, and comfortable tents were pitched on a spot about half a mile to the westward, but here again the medical attendant was doomed to experience a bitter disappointment, for the men got daily worse. By a series of reasoning, which we need not follow seriatim, Dr. Adam was now led to exhibit mercury, that dernier resort in every doubtful or desperate case. One fact, as has been often said, is worth a hundred bushels of argument, and therefore we shall simply state to our readers the results of the Doctor's experience with the drug.

"I accordingly commenced giving calomel in the dose of 10 grains, and ½ grain of opium, three times a day, and continued it till the mouth became sensibly affected, which I think generally happened between the third and fourth day. I need not add how gratified I was to find, that from the moment this occurrence took place, all the symptoms abated. The patients for the first time expressed the relief they experienced from pain and general febrile irritation; and almost immediately the sloughing ceased, and was succeeded by a line of healthy granulations. The change in some of the cases was most rapid and striking, and in almost all, so marked, as to leave no doubt in my mind of its having been brought about by the curative powers of the remedy; and I congratulated myself, that by its means, I was at length enabled successfully to contend with the disease. As far as I can now call to mind, only one case obstinately resisted the powers of the mercury; but what was the issue of it, I am unable to state, having soon after relinquished medical charge of the Division. The turpentine dressings were continued as a local application after the favorable alteration occurred; and in a short space of time, the whole of the sloughs were cast off, and the sores reduced to the state of a simple healthy ulcer. All that appeared necessary now, was to moderate the action of the mineral on the mouth, and support the strength of the patient. But in no instance did ptyalism prove severe, nor was any particular treatment found necessary to effect the latter object. In the restoration of the digestive powers, with a corresponding appetite for food, nature pointed to the most effectual mode of repairing the loss of flesh and strength; and excepting weak Cherayta infusion, which was I believe not always swallowed by the patients, I did not prescribe any further medicine. The men gradually regained their former healthy appearance, and at the period I left the Division, were, with one or two exceptions, in a fair way of resuming their respective duties." 150.

Dr. Adam has had no subsequent opportunity of putting the value of mercury to the test in this disease, but Dr. Guthrie, his successor with the Artillery Division, found it useful both with that, and afterwards with a native corps at Baitool, in the cure of the same kind of malignant ulcer. Dr. Guthrie, however, exhibited the drug more sparingly than our author, and in the form of blue pill, combined with rhubarb. So many circumstances combine to obscure and to vitiate the actual effects of remedies in disease, that much experience and many trials are necessary to establish their value or the reverse. On these accounts, we dare hardly pronounce that Dr. Adam has adduced convincing proofs of the real powers of mercury in sloughing ulcer. The question must yet be considered *sub lite*, but we are sure that the profession, and our Indian brethren in particular, will feel much indebted to Dr. A. for directing their attention to the remedy.

We now arrive at the paper of Mr. Leslie, on the same disease, though occurring at a different time and in a different portion of the Indian territory. The subject is one of such practical importance, and Mr. Leslie's contribution differs so much from the preceding author's, that we should not be doing our duty by the public if we slurred it over in a hasty or superficial manner.

In 1823, the 65th Regiment of B.N. was raised for general service at Dinapore, and in December, 1824, our author was appointed to the medical charge of the corps. At that time, the average number of patients in hospital was seventy, the majority eruptions and slight intermittents. In February, 1825, the regiment marched to Barrackpore, when much sickness prevailed, chiefly obstinate bowel-complaints and intermittent fevers, with some few cases of extensive sores, but none of a malignant character. In July they left Barrackpore for Prince of Wales's Island, at which Presidency they arrived in the middle of August; and, on the 1st of September, the number of sick in hospital was 123, of which cases 10 were ulcers. The latter cases rapidly increased, and in the month of November four died. In December they amounted to ninety, the greater number of gangrenous character, and fresh cases continually occurring, a committee, composed of all the medical officers at the Presidency, assembled in January, and agreed on a report to Government, recommending the removal of the sick to an elevated station, and the substitution of flour, sugar and sago, for their ordinary rations, rice and *dholl*. Beer and wine were also to be expended at the superintending surgeon's discretion.

"These measures were speedily adopted, and in the end of January, one hundred cases of ulcers were removed to two spacious temporary buildings, erected for the purpose on the summit of one of the hills. On the removal, the men generally expressed themselves as feeling better; and several of the sores which had been sloughing for weeks in the hospital below, became clean, and were ultimately cured. The more severe cases, however, seemed to sink more rapidly than before; and in February 11 died, the mortality of the preceding month having been nine. For some weeks after the removal of the sick, no fresh cases had sloughed; but about the beginning of March, ulcers now commenced in several of the cicatrices, rapidly progressed, attended with much fever, great pain, and very quick pulse, and in several cases proved fatal, after extensive gangrene had taken place. No fresh cases being admitted to hospital during February from the lines, the total number in hospital being consequently much diminished, on the weather proving unfavorable, and the supply of water being deficient, and several severe cases of bowel-complaints occurring, the establishment on the hill was finally broken up in April; and the remaining cases, now reduced to 30, returned to the line hospital.

"Five legs were amputated in March, two of which cases died: four were removed in April, of which two also were lost. The cases of ulcers now in hospital being only about 20, and free from any unfavourable character, the malignancy of the disease appears to have wholly subsided—(May 20th.)" 154.

After these general and preliminary remarks, Mr. Leslie proceeds to consider, seriatim, the topography of the island in the immediate vicinity of the cantonments, the medical history of the disease, its etiology, and, finally, its treatment.

Fort Cornwallis is situated at the extremity of a tongue of land, stretching to the opposite Malay coast, from which it is distant from one and a half to two miles. It is bounded to the westward by a range of high hills, which form a triangular and level area 20 to 30 square miles in extent, and comprising the town and military cantonments, the former extending for about a mile along the shore. The outlet of a small river less than a mile from the fort, the inundations of the neighbouring ground by the tide, the general abundance of jungle over the area, and the collection and stagnation of water after heavy rains, conspire to make the tract in question peculiarly favourable, one would think, for miasmal exhalations. Nearly three miles inland from the fort, a space, above a square mile in extent, has been cleared for parade, and in its vicinity are the European and Sepoy hospitals, both in a very decayed condition; and although raised seven feet from the ground, the floors are in such a state as by no means to protect the inmates from the effluvia from beneath. The vicinity of the hospitals is very damp, and the same may be said of the lines in which the men are lodged, whilst the latter were, till lately, very much crowded. So much for the topography of the spot in which the disease broke out, and it does most certainly appear not ill adapted for the rise and spreading of any epidemic.

### MEDICAL HISTORY.

When the cases first began to excite serious apprehension, the following symptoms were observed.

"A sore which had for a period been indolent, or even disposed to heal, would begin to ulcerate, some parts becoming excavated, the discharge vitiated, the edges becoming elevated and everted, and in some cases undermined. At this period, unhealthy sores generally showed a disposition to become circular and very painful. In this state they would continue for a time, deriving perhaps slight temporary benefit on each change of application, but on the whole becoming worse, and more extensive sloughs from time to time were thrown off, the exposed surface appearing clean for a day or two, and then assuming a foul appearance. Emaciation was progressive, but no very marked febrile affection was constantly attendant. At length, frequently after weeks of suffering, the patient died exhausted, without much change in the sore: in other cases, sphacelus to some extent occurred before death took place.

"2. After some weeks experience of the disease, cases of a different type occurred.

"A sore would occasion great pain, and on examination a small black spot would be observed in the centre; or in other cases, the slough for several days after its appearance would continue of a light greyish color, but uniformly advancing. These cases were almost always attended with severe constitutional disturbance, hot dry skin, urgent thirst, and frequent pulse. The slough in a short period covered the whole sore, and rapidly extended, in several cases, from the foot or ankle to nearly the knee, before death occurred: the whole limb, in many cases, resembling a piece of charred wood, there being little or no discharge." 156.

The symptoms in all cases were not quite uniform. In some, the patients lived until after the sloughs had been thrown off; in others, no such separation of the dead parts took place. In some, the general fever kept pace with the local complaint, and the patient became comatose before death; in others, the senses were retained to the last, and nothing but extreme prostration of bodily power was observed. Sometimes ulcers commenced with the characters first described, and ended with those of the second type.

"3. At a subsequent period, a third species of sore appeared, affecting the cicatrices of ulcers, which had either entirely or very nearly healed. Ulceration would commence, and go on for days, exciting perhaps during that period, not much attention on the part of the patient, (whose general health for a time did not appear to suffer,) but steadily resisting every application, and becoming progressively worse. A purplish livor of the surrounding integuments was usually present in these cases, but for a time but little pain was experienced; nor, as before remarked, did constitutional symptoms shew themselves till the sore had made some progress. Diarrhœa then supervened, the dejections were fetid and loose, the patient became feverish, and the pulse frequent. In several cases of this species, sphacelus, to a considerable extent, occurred before death: in one case, the limb was successfully removed." 157.

Mr. Leslie observes that, although insurmountable difficulties arose in forming a decided opinion on the constitutional symptoms, yet he has no hesitation in affirming that the disease was *not* contagious. If this be true, the disease was certainly different from most of the epidemics of hospital gangrene, of which any published accounts have been given. Where the sores occurred in the vicinity of tendons, they almost invariably sloughed to some extent, and this was the case with the tendo Achillis in several instances. The sores were usually situated on the ankles, heel, or lower third of the legs, but three proved fatal on the thigh, and one man died from gangrene in the groin, originating in bubo. The bones in many cases ulcerated, and not unfrequently exfoliated. In four patients mental derangement supervened; the first occurred after amputation, lasted for three weeks and then subsided; but the three others underwent no operation, and continued insane. In two of the latter, however, the hallucination appears to be gradually giving way.

### ETIOLOGY.

It appears that in this, as in most other maladies, a variety of causes conspired to produce the pernicious effect. In the first place, it is well ascertained that natives of the Western provinces invariably suffer severely with bowel-complaints, fevers, and foul sloughing ulcers, on their removal to our Eastern settlements. In the present case, this already powerful source of disease was augmented and enforced, by the circumstance of the greater part of the newly-raised regiment consisting of young men, many, indeed, mere boys of 16 or 18 years of age, who scarce possessed a coverlet on which to lie, and thoughtlessly exposed themselves to the vicissitudes of the climate, and indulged in fruit and cheap, unwholesome fish. The rations, too, for several months, were extremely deficient, and the men, in consequence, were obliged to subsist on rice alone, with indifferent vegetables, dressed in oil, without a due proportion of spice. The state of the hospitals and medical topography of the cantonment we have fully noticed already, and need not advert to them any further than merely to observe, that they necessarily formed very powerful links in the chain of causation. It is a curious, but not uncommon fact, that the improved rations of flour, sugar, and sago, allowed by Government, were not received with any thing like gratitude by the men. The sago they would not generally use, and, at length, its distribution was obliged to be restricted to those who consented to employ it. It is constantly thus, that the benevolence and humanity of medical men are repaid by distrust and ingratitude in other climes than the "land of the sun," and by other more civilized patients than the ignorant and prejudiced Hindoo!

### TREATMENT.

The following are the author's remarks on the local applications, and they shew most amply, in our humble opinion, that however useful each or all of these may occasionally be, they are quite as often of no avail. We have seen ourselves the powers of the compound tincture of benzoin in checking rapid and destructive sloughing, and the balsam of Peru has also proved of great service in epidemic hospital gangrene. Mr. Leslie, however, employed both, and, it seems, without experiencing that marked good effect which the same means have had in other hands. This is one of those occurrences in medicine, which should teach us to beware of generalizing too boldly from the limited observations of individuals.

"In describing the first appearance of the disease, it was remarked, that sores became foul, and sloughed, with but little constitutional disturbance: on this account, and at this period, unremitting attention was paid to the application and effect of local remedies: of these, generally and extensively used, may be enumerated the following. Poultices of *papeeta*—of charcoal with *taree.** Bals: of Peru.—Tr: Benz: et Aloes Comp.—Tr. Myrrhæ, Terebinth: Venet. Lin: Terebinth. Ung: Hyd: Nit: Ox: rub: resinos. Lot: Sulph. Acet: cupri. Nit. argenti. Acid Nit. dilut. Lot. nigra, &c.

"As to the utility of these, a very few remarks will suffice. Poultices were not useful. The Bals. of Peru failed in producing a healthy action in the sores, though it certainly possessed considerable power in correcting their fetor. The different tinctures did no good; though, mixed with a proportion of turpentine, they answered better than the ointments, which were very sparingly used, excepting resinous or common ointment, placed above, but dipped in some other dressing.

"Regarding local applications, none on the whole appear superior to the common lotion of nitric acid diluted; this, however, was borne stronger than it is generally directed to be applied, (dr. iss. oz. xxiv.) In particular cases, the Terebinthine liniment, black wash, and solution of lunar caustic, as also the same in substance, were advantageously employed." 163.

Two other applications, certainly of very opposite character, were employed, although not to a sufficient extent to set the results above suspicion. These were, the steam of hot water, and the undiluted nitric acid. The former in the cases in which it was tried seemed to give relief by its soothing effects; the latter did service in some cases, completely failed in others, and in a very few, a single application arrested the sloughing, and the sores healed. Of the general treatment we must inform our readers in the author's own words, as we cannot easily abridge the passage.

"It now remains to mention the means had recourse to as general remedies: these necessarily varied with the type of the disease, and at different periods various remedies became requisite. As already mentioned, at the commencement the sloughing process was slow, the patient lingered long, and seemed at last to be worn out with constant suffering. To obviate these symptoms, the secretions in general were attended to, the bowels kept open by the purgatives in common use, and no more nutritious diet was advised. During the months of December and January, the disease, which had been gradually becoming more extensive, now also assumed a more serious aspect. Gangrene, when it took place, was extremely rapid in its progress; these cases being attended with a frequent small pulse, and dry harsh skin; frequently little or no discharge took place from the sore; occasionally a few vesications formed, when the parts were dead underneath. Several of these cases sank without diarrhœa, though others suffered severely from this symptom: in not a few it only occurred a very few days before death. During the greater part of the above period, no medicines or remedies, either general or local, appeared beneficial. The unfavourable local circumstances have already been adverted to. Of the remedies used are to be mentioned, mild purgatives, opium in various forms and combinations, (rendered urgently necessary by the severity of the pain,) and the warm bath, which certainly gave relief, and for a time appeared to lessen the dryness of the skin. In January, beer was allowed to several of the worst cases, some of whom relished it; and it seemed to protract the life of some, who eagerly took it.

* "The fermented sap of the palm, called the Tar or Toddy-Tree."

About two-thirds of a bottle, given twice, at noon and bed-time, daily, was the usual quantity: few wished to take more. Those who were so weak as to be unable to swallow so much liquid, had wine, from two to three glasses daily.

"Some of the cases certainly derived advantage from these means; but they never produced the good effects which were anticipated by the Committee.

"The objection of the natives to post-mortem researches is well known, and up to this period only one body had been examined. Feeling, however, the imperious necessity of this measure, the bodies of two men who died in February of rapidly progressing gangrene, were examined. In one, as in the first case, much structural derangement of the alimentary canal was discovered; but in the other, no organic lesion found. In both, during life, the symptoms had displayed much resemblance. In subsequent examination, however, the mucous membrane of the alimentary canal, particularly of the colon, seemed to bear the onus of disease. From this period (February) calomel combined with opium was more systematically administered, though certainly for a time with but little success. The form which appeared to answer best was a combination of Dover's powder, seldom less than half a drachm, to 4, 6, 8, or even 10 grs. of calomel. This dose was given at bed-time, and if the pain was urgent, opium was also given during the day, sometimes with calomel, blue-pill, ipecacuanha, or antimonial powder, and sometimes without any of these. In several cases the mouth became affected, in some followed by permanent, in others only temporary benefit, and in a third set with no very marked effect, either favourable or otherwise. The purgatives in general use were jalap, rhubarb, castor oil, and infusion of senna: beer and wine were more sparingly administered, and the infusion of gentian with sulphuric acid substituted instead. Those who derived benefit from its use, of course were allowed to continue it. Towards the end of February, a case was sent from the line hospital to the hill, which afforded a favourable opportunity of trying the effects of it. The patient was stout and robust, the sloughing sore situated exactly on the ligamentum patellæ, and the joint felt somewhat hot and swollen: much pain was experienced. 20 ozs. of blood were taken away, the coagulum of which was firm, but showed no buff. In three days the sore became clean, and the man recovered. Several other cases, in which the febrile action ran high, were moderately bled, (to the extent of 8, 10, and 12 ozs.) in none with any bad effects: even in some who derived no ultimate benefit, temporary relief was obtained from a painful sense of throbbing. In the use of this remedy, though likely to prove beneficial at the onset, where much febrile action is present, and before the patient's strength is reduced, the greatest caution must be observed. Impressed with the belief, that the constitutional symptoms were such as to forbid all prospect of advantage from operation, no attempt had been made to save any of the cases by this measure until March, when a case occurred, attended with less constitutional disturbance than usual, and in which the limb was removed, and the man was discharged well in 23 days. As the operation was borne well, it was resolved to give the chance to another case then in hospital, and which was most evidently sinking, from inability of the constitution to heal so extensive an ulcer as existed. Contrary to expectation, this man survived: and three more limbs were removed in the course of the month. One of these operations was done, however, solely in compliance with the urgent supplication of the patient, and proved unfavourable. One other case also died. In both, extensive disease existed. From causes already detailed, the establishment on the hill was finally removed in April; and after the return of the cases to the line hospital, four legs were amputated in this month, two of which recovered. These operations, except perhaps the first, which promised success, were done under exceedingly unfavourable circumstances: nevertheless five out of nine, or rather, out of eight, having recovered, proves, (in one the stump healed by the first intention, and the man speedily regained flesh and strength,) that this measure, without hesitation, and under like circumstances, may be adopted with a reasonable prospect of success." 168.

It appears that amputation was performed in nine cases, five of which did well, certainly not a discouraging result.* It does, however, seem strange, and, to our minds, contrary to every thing like principle, that amputation should succeed in this disease. Here we see malignant ulceration grafted on the simplest wounds and even scratches, whilst it does not succeed the larger and more dangerous injury inflicted by the knife. It is just one of those puzzling facts in medicine, that contribute to its uncertainty and its empiricism. We have now concluded our analysis of Mr. Leslie's observations, for the remainder of the paper which is occupied by individual cases and numerical returns, we need not particularly notice. Every contribution to the history of so formidable a malady as that under consideration must be read with interest by our brethren, particularly by those in military, naval, or colonial service, whose hap it may be to encounter "the foul fiend" in all his horrors. In this country we are generally free from the inroads of these pestilential distempers :—

> "Flaming carbuncles and noisome sweat,
> "And clammy dews, that loathsome lice beget;
> "Till the slow-creeping evil eats his way,
> "Consumes the parching limbs, and makes the life his prey."

### VIII.

* The total number of cases admitted into Hospital between September 1st and April 30th, 1826, a period of eight months, was 281, of which died 40, being one to seven and one-fortieth, or fourteen and one-fourth per cent. very nearly.

